# Dual-functional communication and sensing antenna system

**DOI:** 10.1038/s41598-022-24812-3

**Published:** 2022-11-27

**Authors:** Tanjir Alam, Michael Cheffena, Eva Rajo-Iglesias

**Affiliations:** 1 Faculty of Engineering, Norwegian University of Science and Technology (NTNU), 2815 Gjøvik, Norway; 2 Department of Signal Theory and Communication, University Carlos III of Madrid, 28911 Madrid, Spain

**Keywords:** Electrical and electronic engineering, Electronic and spintronic devices

## Abstract

In this article, a dual functional antenna system for communication and sensing applications is designed on a single substrate having a common input port. The main motivation behind the proposed design is to build an antenna system, which can work as an antenna sensor and also can communicate over a fixed band not related to the resonance frequency shifts of the antenna sensor. The dual functionality of the system has been achieved by designing a frequency selective multipath filter (FSMF). The FSMF has an input port and two output ports. Both the communicating and sensing antennas are integrated on the output ports of the FSMF. The FSMF ensures minimum effect of the antennas on each other’s performance. The performance of the antenna sensor is first shown by characterizing different standard substrates with ${\varepsilon} _r=2.2$ to 6.15 and then demonstrated for ice and water detection. The communicating antenna is used for Wi-Fi (2.45 GHz) applications. The simulated and measured results of the dual-functional antenna systems are in good agreement. The proposed design is fabricated on a PCB with ${\varepsilon} _r=3.5$ and $\tan \delta =0.0027$ with an overall size of $86.7\times 26\times 0.8\,\text {mm}^3$.

## Introduction

Antennas are are key components of wireless communication systems. By perturbing their electric field or functionalizing their surface utilizing sensing materials, antennas also have massive potential in sensing applications. Antennas for sensing applications, often referred to as antenna sensors, are gradually gaining much interest compared to the other microwave sensors.

Based on their signal transmission and reception properties, wireless antenna sensors can be broadly classified into two main categories: active and passive antenna sensors. The term “active” indicates the capability of an antenna to transmit/ receive electromagnetic waves, while the word “passive” emphasizes the capability of backscattering the incident electromagnetic wave. Radiofrequency identification (RFID) tags are an example of passive antenna sensors. RFID tag-based passive antenna sensors explore the backscattering principle of the forwarded electromagnetic waves. In this case, a reader does both the generation and reception of the signal, and dipole-based RFID tags are just used to backscatter the transmitted signal^1^. The amplitude and phase of the backscattered signal depend on the properties of the medium surrounded by the RFID tag. Based on this principle, RFID tag antennas were used for several sensing applications^1–3^.

Graphene printed RFID tag antenna coated with graphene oxide (GO)^1^ can be used for humidity sensing applications. The amount of water uptake at various humidity conditions can change the relative dielectric permittivity of the GO. This property can ensure a change in the resonance frequency of the GO-coated RFID tag by changing the antenna impedance. Also, depending on the different humidity conditions at the vicinity of the antenna, the RFID reader can observe changes in the phase of the backscattered wave. In the case of active antenna sensors, the properties of the antenna (resonance frequency, input reflection coefficient, and transmission coefficient) change as it transmits or receives the signal. Microstrip patch antenna (MPA) sensors can become a promising choice at microwave frequencies due to the several advantages like wireless monitoring ability, real-time measurement, low-cost planar design, small volume, robustness, etc. These advantages can make the MPA sensor a very efficient and cost-effective choice as an active antenna sensor. Additionally, its higher operational frequency miniaturizes the size and reduces the volume of the overall system.

The resonance frequency of an MPA is dependent on the substrate permittivity and dimension of the patch. These two parameters are sensitive to the temperature, which generates the possibility of using MPA as a temperature sensor^4^. Using a multi-resonant system, simultaneous sensing of three parameters (e.g., temperature, pressure, and humidity) is also possible^5^. The multiple resonances can be achieved with the help of complementary split-ring resonators (CSRR)^5^. The dimension of the CSRR defines the resonance frequency. Antenna sensors are also used for structural health monitoring (SHM) applications^6,7^. The decomposition process of a structure over time is a natural phenomenon. However, it can become faster due to rapid weather changes and other environmental calamities. Hence a real-time health monitoring system for these structures is essential. Using antenna sensors real-time SHM systems to monitor strain, temperature, and moisture were widely implemented^8–11^.

**Figure 1:**
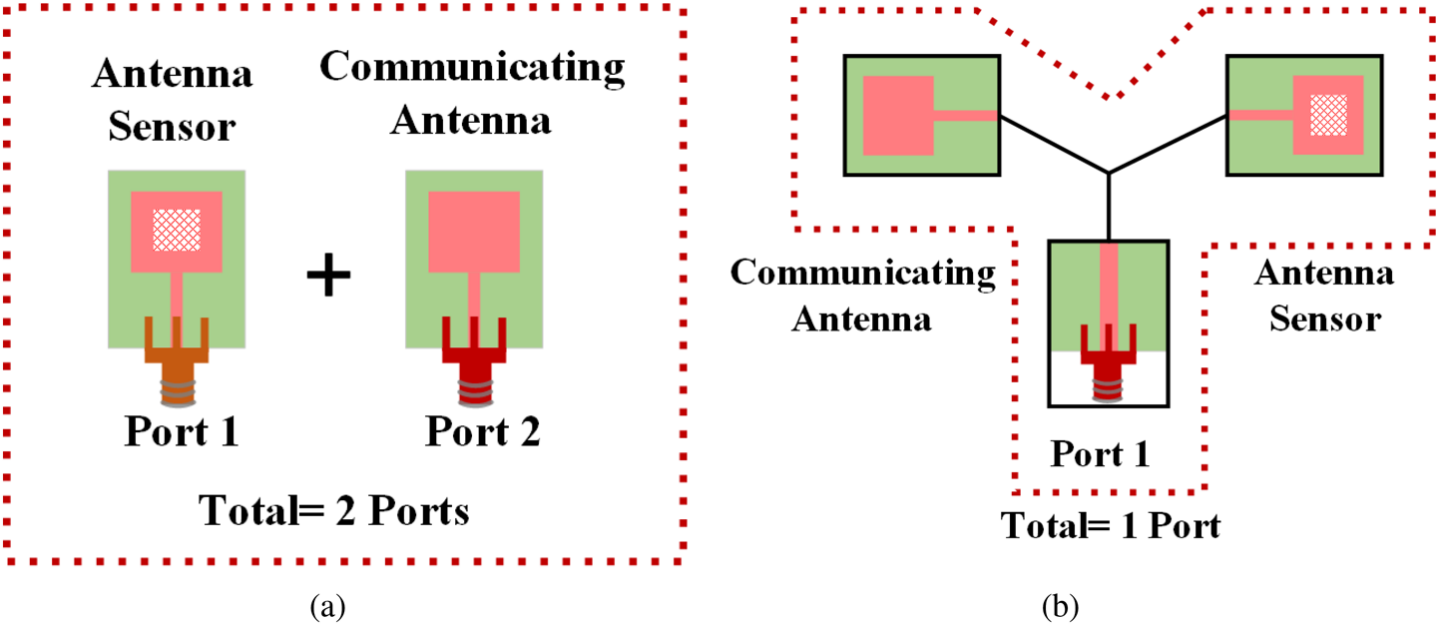
(**a**) Separate antenna and antenna sensor on a substrate requiring two different ports, vs (**b**) our proposed design with a single port.

**Table 1:** Comparison with state-of-the-art.

Parameter sensed	Measuring parameters	Operation (Sensing/communication)
Temperature^4^	Resonant frequency	Sensing
Methanol^12^	Input reflection coefficient	Sensing
Temperature, pressure, humidity^5^	Input reflection coefficient	Sensing
Air pressure^13^	Resonant frequency	Sensing
Deformation^14^	Input reflection coefficient	Sensing
Glucose^15^	Transmission coefficient	Sensing
Tumor^16^	Input reflection coefficient	Sensing
Ethanol^17^	DC output voltage of the rectifier	Sensing
Humidity^1^	Backscattered signal	Sensing
Water and ethanol in oil^18^	Input reflection coefficient	Sensing
(*This work) Ice/water, dielectric materials ${\varepsilon} _r=2.2$ to 6.15	Input reflection coefficient	Sensing and fixed band communication

Volatile organic compounds (VOCs) with lesser molecular weights are harmful to human health. VOCs’ selective detection can be achieved utilizing an MPA coated with a sensitive material (i.e., a material that changes its Physico-chemical properties in response to the accumulation of a target analyte). This type of sensor can provide an efficient, fast, responsive, and cost-effective solution for detecting hazardous air pollutants like VOCs^12^. In^12^, a substrate integrated waveguide (SIW) based antenna sensor was used with a polyindole (PIn) film for the detection of methanol. The film was deposited over the higher electric field region of the cavity. This PIn film provides higher selectivity towards methanol. The addition of the film can drastically reduce the cross-selectivity issues of the antenna sensor as the film has a higher affinity towards methanol compared to other VOCs. In the literature, antenna sensors for other different applications including chemical substances detection^19,20^, drug detection^21^, breath monitoring^22^, biomedical applications^15,16^, air pressure sensing^13^ or deformation detection^14^, were also presented. Recently a flexible rectifying filtering antenna^17^ was used for ethanol detection, which uses a resonator with a microfluidic channel. The system was wirelessly powered and was used as a bracelet to detect the presence of ethanol in hand sanitizers. Recently, another contactless antenna sensor with an artificial magnetic conductor (AMC) array was presented to monitor oil quality^18^.

However, existing antenna sensors were solely used for sensing purposes. A comparison of the existing works on antenna sensors is summarized in Table 1. Depending on the detection of a target analyte or changes in any physical parameters in the vicinity, an antenna sensor changes its resonance frequency, which makes the antenna incapable of communication over a fixed frequency bandwidth.

One possible solution can be the usage of multi-band antennas, where different frequency bands can be used for communication and sensing applications. However, the presence of the sensitive material on the antenna surface or perturbation of the electrical field can affect both the frequency bands. That means that the effect of the frequency shift of one band can also impact the other operational frequency bands^5^. Hence, becoming problematic for a communicating antenna to operate over a fixed frequency band. Another solution could be using an adaptive matching network^23^, which can adaptively match the change in the antenna impedance due to changes in the sensed physical parameters. Thus, the antenna sensor can remain operative over the fixed bandwidth. Unfortunately, these types of circuits are complex and can add significant complexity and cost to the antenna sensor system.

**Figure 2:**
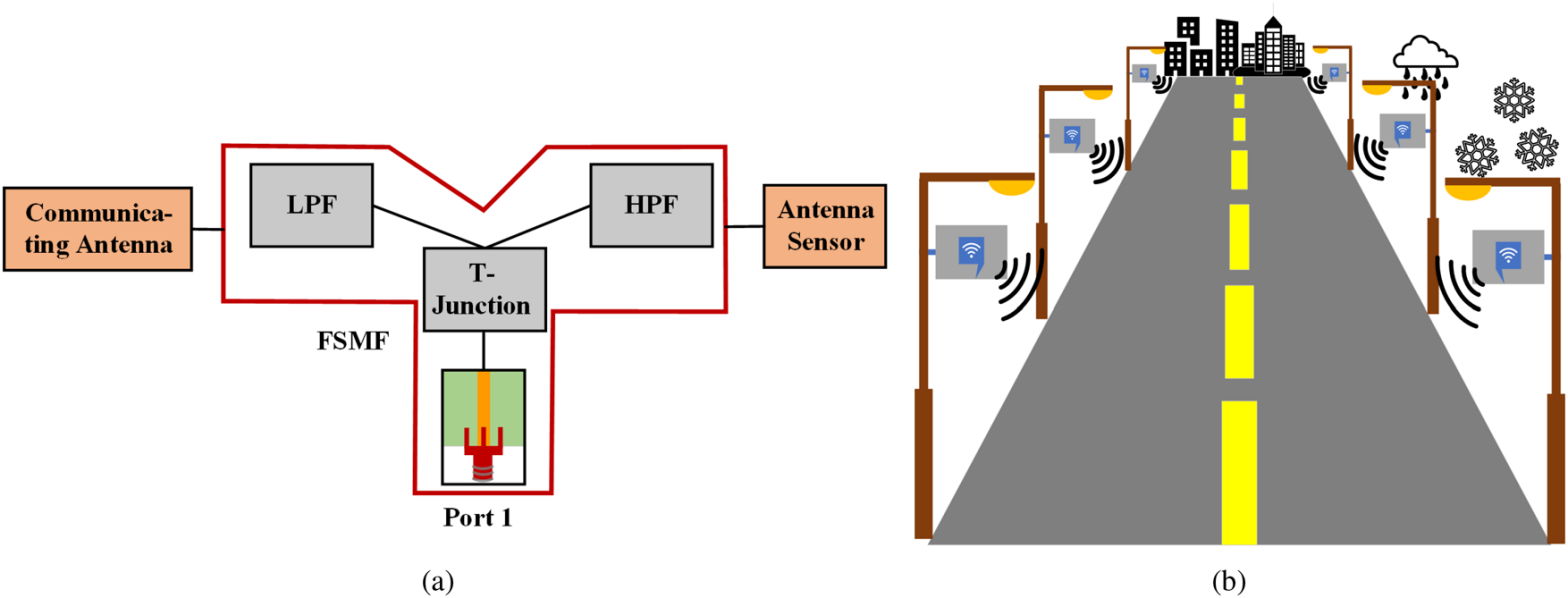
(**a**) The design overview of the proposed system. (**b**) Wi-Fi access points around the roadway infrastructures.

**Figure 3:**
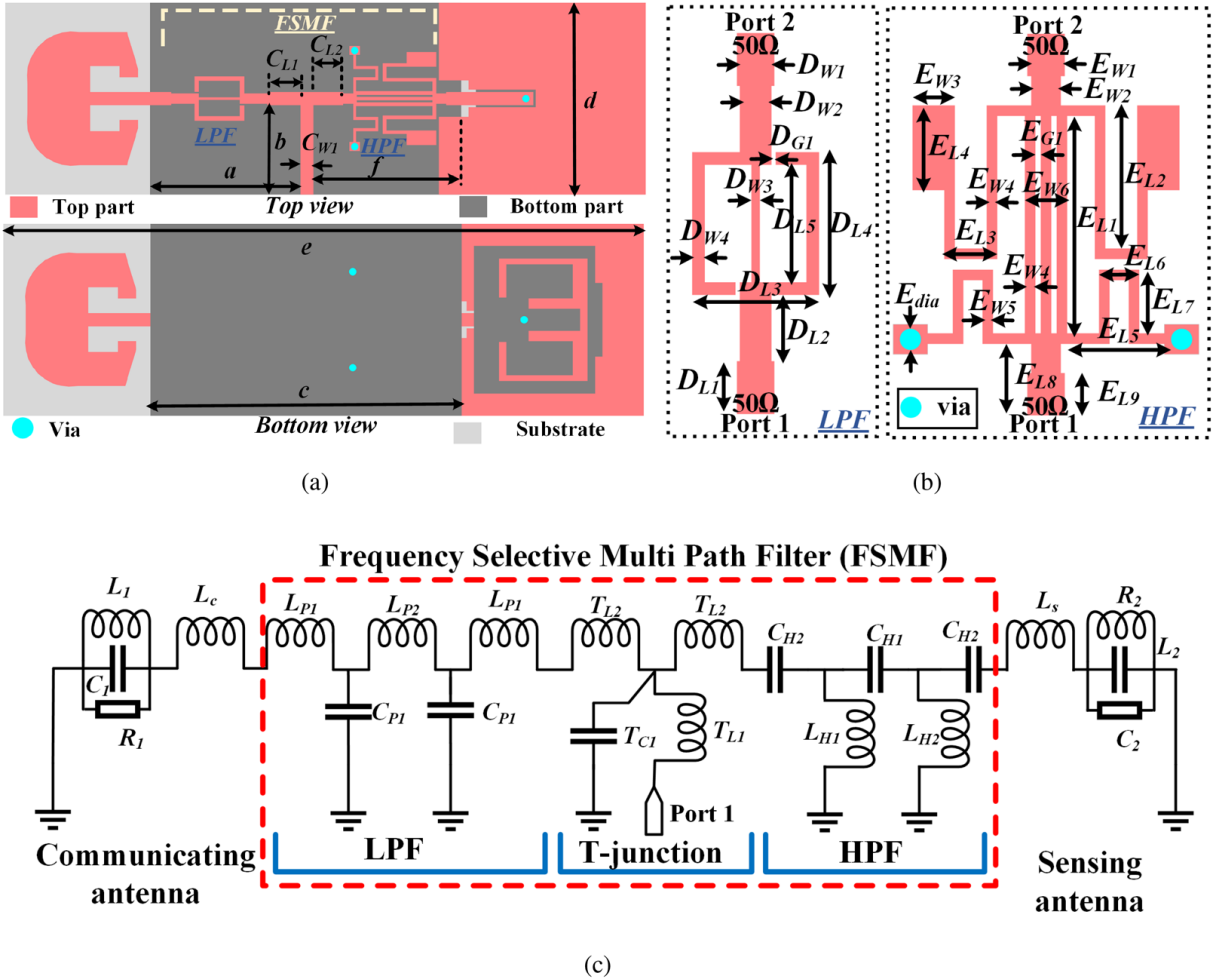
(**a**) Top and bottom view of the proposed dual-functional system, (**b**) dimensions of the LPF and HPF integrated into the FSMF of the proposed system, and (**c**) Equivalent circuit of the proposed dual-functional system.

Moreover, no significant studies have been done on the simultaneous use of an antenna as a sensor and a communicating device (i.e., a dual functional antenna system). This creates the problem formulation and main objective of our proposed work.

In this work, we have proposed a novel concept of using an antenna system as a sensor as well as a fixed band communicating antenna without affecting each other’s performance in the same substrate using a single port. The proposed dual-functional design reduces the number of port requirements for sensing and communication application in a single substrate from 2 to 1. As shown in Fig. 1a, a separate antenna sensor and fixed band wireless communication system on a single substrate would require a minimum of 2-ports (one for the antenna sensor and one for the communicating antenna). Instead, our proposed design (Fig. 1b) uses a single port for both applications. This single-port dual-functionality is achieved by using a frequency-selective multipath filter (FSMF).

The FSMF creates two separate output paths from the same feed network. A low pass filter (LPF) and a high pass filter (HPF) are connected on the two output paths of the FSMF. The dual-functionality of the proposed system is achieved by integrating a communicating antenna and an antenna sensor on the output ports of the FSMF. The complete design overview of the proposed system is shown in Fig. 2a. Our proposed design provides the following main contributions:

- It can simultaneously perform as an antenna sensor and a fixed band communicating antenna having the same input port.
- It reduces the total port requirement from 2 to 1.
- It eliminates the requirement of using a matching network, which adds losses and system complexity, thus increasing the overall cost.

The fixed band communicating antenna of the proposed dual-functional system is proposed for Wi-Fi application at 2.45 GHz, and the narrowband antenna sensor is used for ice/water detection. Additionally, the performance of the antenna sensor is further validated to sense different materials (substrate blocks) of permittivity ${\varepsilon} _r=2.2$ to 6.15. However, the concept can be used for any other simultaneous sensing and communication applications.

**Figure 4:**
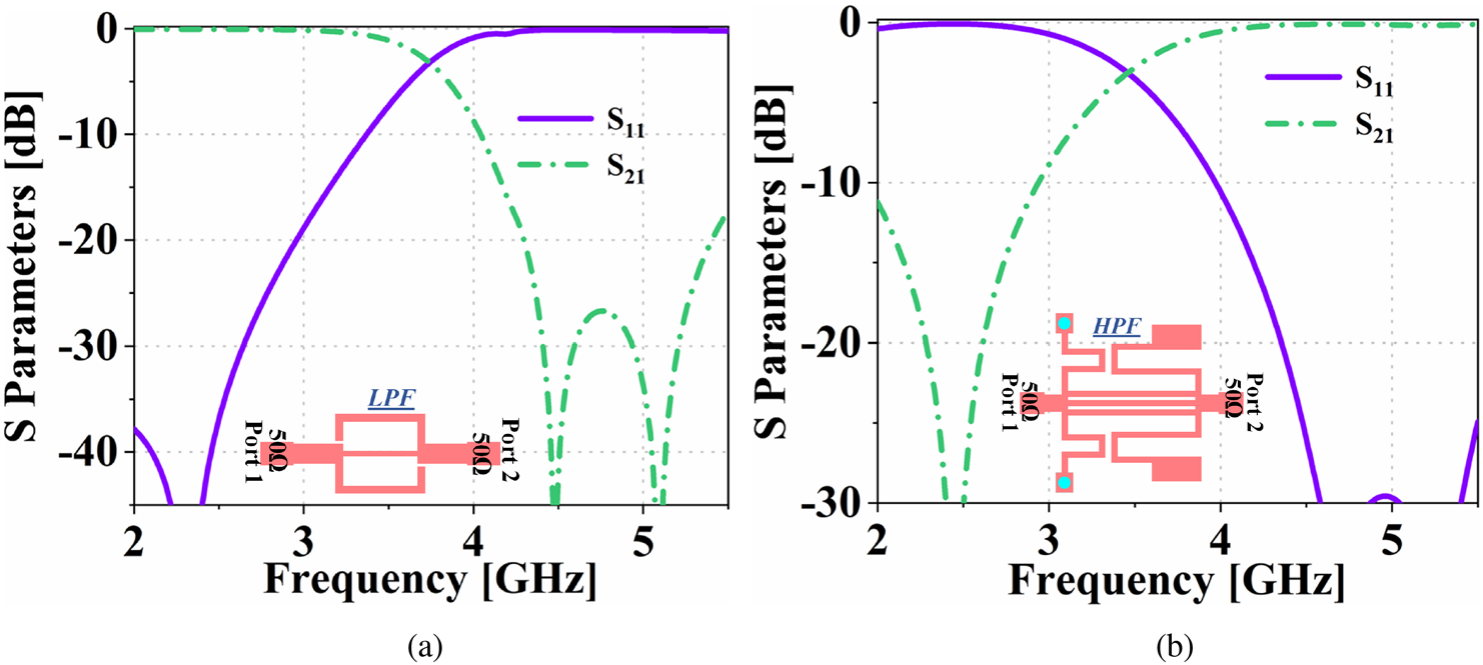
S-parameters of the the (**a**) LPF and (**b**) HPF, integrated into the FSMF of the proposed system.

**Figure 5:**
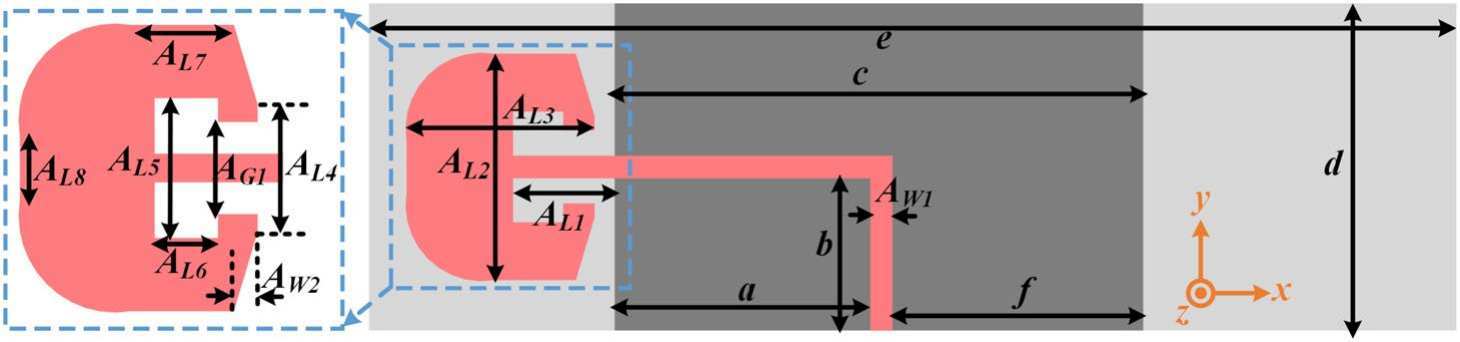
Proposed antenna for fixed band communication application.

**Figure 6:**
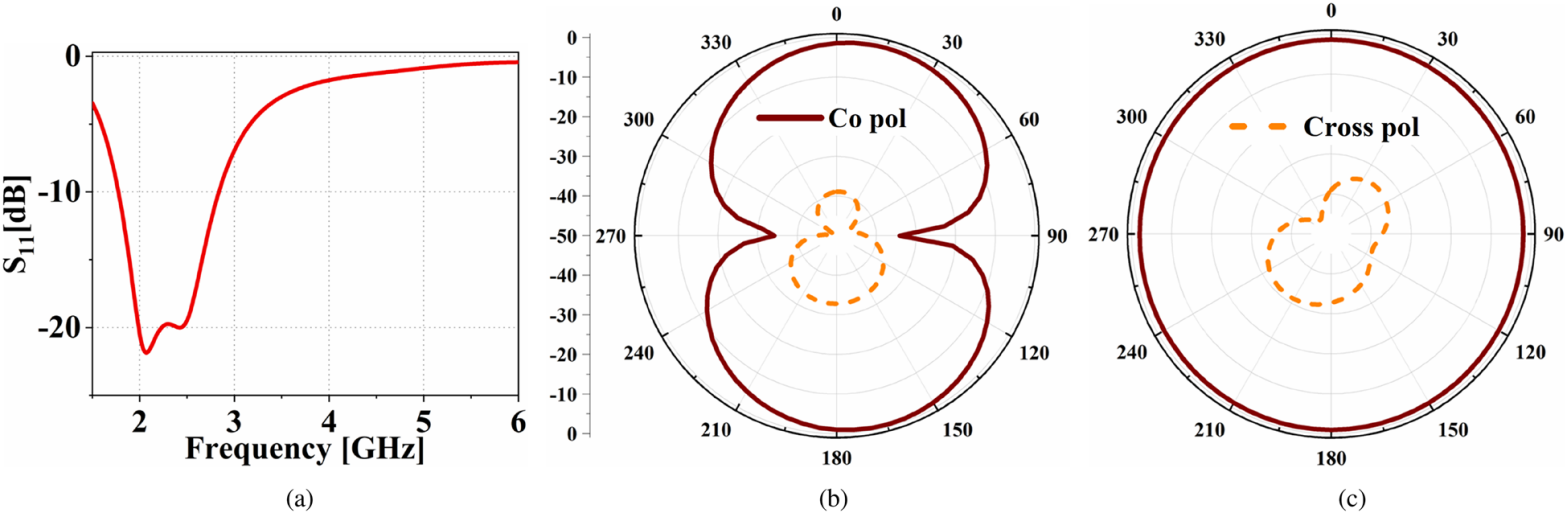
(**a**) Input reflection coefficient of the proposed fixed band communication antenna.The simulated radiation pattern of the antenna at 2.45 GHz in (**b**) $\hbox {phi}=0^{\circ }$, (**c**) $\hbox {phi}=90^{\circ }$ planes.

Ice accretion can cause additional problems in a wireless communications system (e.g., remote GPS antennas, base stations, radome of an aircraft, mobile communication systems, radio broadcasting antennas, etc.). For example, ice deposited on the radome of Wi-Fi access points installed in open environments across several parts of a city (e.g., railway stations, airports, playgrounds, around the roadway infrastructures, etc.) can cause distortion of the signal by increasing the transmission loss, which reduces the system performance and the covering range. It also affects base stations in the same way by reducing the signal-to-noise ratio, which mainly occurs due to the increased path loss. Additionally, the icing on the blades of a wind turbine can reduce its efficiency by affecting its aerodynamic profile. Furthermore, if not timely detected, accretion of ice on critical surfaces like runways, roads, industrial equipment may cause several safety issues. Thus, detection of ice becomes very important for safe operation in the aforementioned application scenarios. In our proposed design, for the efficient performance of the system, the sensing plane and the radiator patch of the Wi-Fi antenna are kept on the opposite side of the substrate. As can be seen from Fig. 2b, which represents roadside Wi-Fi access points. The accumulation of water/ice mostly happens on the top side, while communication with the passengers occurs on the other side.

## System configuration

The overall system includes an antenna for fixed-band communication, an antenna for sensing application, and an FSMF. The design details of the proposed dual-functional system is shown in Fig. 3a, where, $C_{L1}=4.5$, $C_{L2}=4$, $C_{W1}=1.77$, $a=20.4$, $b=12.1$, $c=42.2$, $d=26$, $e=86.8$, $f=20$.The FSMF shown in Fig. 3a includes a LPF and a HPF on its two output path. The filters are designed by following the methodology proposed in^24–28^. The dimensions of LPF and HPF integrated into the FSMF of the proposed system are shown in Fig. 3b, where, $D_{W1}=1.77$, $D_{W2}=1.5$, $D_{W3}=0.4$, $D_{W4}=0.6$, $D_{L1}=2.5$, $D_{L2}=3.1$, $D_{L3}=6$, $D_{L4}=6.8$, $D_{L5}=5.6$, $D_{G1}=0.2$, $E_{W1}=1.77$, $E_{W2}=1.4$, $E_{W3}=2$, $E_{W4}=0.5$, $E_{W5}=0.4$, $E_{W6}=2$, $E_{L1}=10.4$, $E_{L2}=6.8$, $E_{L3}=2.5$, $E_{L4}=4$, $E_{L5}=5$, $E_{L6}=1.9$, $E_{L7}=3$, $E_{L8}=3.4$, and $E_{L9}=2$, (all in mm). S-parameter responses of the individual LPF and HPF are shown in Fig. 4. The equivalent circuit of the proposed dual-functional system is represented in Fig. 3c, where the capacitance and inductances associated with the T-junction are represented by $T_{C1}$, $T_{L1}$, and $T_{L2}$. In the LPF equivalent circuit, $L_{P1}$ and $L_{P2}$ represent the series inductances associated with the stepped microstrip line, while $C_{P1}$ represents the shunt capacitances related to the open stub lines. Similarly, in the HPF equivalent circuit, $C_{H2}$ represents the capacitances associated with feed line discontinuities, and $C_{H1}$ represents the total equivalent series capacitance due to the slots of gap $E_{G1}$. At the same time, $L_{H1}$ and $L_{H2}$ represent the total equivalent inductances associated with the shunt microstrip lines. The lumped parameter values evaluated using Keysight ADS of the circuit model presented in Fig. 3c are $L_{P1}$=0.5 *nH*, $L_{P2}$=0.83 *nH*, $C_{P1}$=1.54 *pF*, $C_{H2}$=0.73 *pF*, $C_{H1}$=0.54 *pF*, $T_{L1}$=0.50 *nH*, $T_{L2}$=1.81 *nH*, and $T_{C1}$=0.98 *pF*. Each RLC^29^ tank circuit represents the resonance frequency of the antennas. $L_C$ (0.5 *nH*) and $L_S$ (1.82 *nH*) represent the inductances of the communicating and sensing antenna feedlines. The RLC tank circuit connected at the output port of the LPF represents the communicating antenna, where the resonance frequency and the quality factor of the communicating antenna are defined as $f_{rCA}=1/2\pi \sqrt{L_1C_1}$ and $Q_{CA}=2\pi f_{rCA} {R_1C_1}$. Similarly, the sensing antenna is connected at the output port of the HPF. The resonance frequency and the quality factor of the sensing antenna are defined as $f_{rSA}=1/2\pi \sqrt{L_2C_2}$ and $Q_{SA}=2\pi f_{rSA} {R_2C_2}$. The values of the RLC tank circuits are $R_1$=60.28 $\Omega$, $R_2$=99.97 $\Omega$, $L_1$=0.71 *nH*, $L_2$=0.5 *nH*, $C_1$=6.11 *pF*, and $C_2$=2.05 *pF*. The equivalent circuits of the LPF and HPF are presented before the RLC tank circuits. The communicating antenna is connected at the outport port of the LPF. Hence, the LPF is designed such that it has a maximum matching near about 2.45 GHz. Similarly, the sensing antenna is connected at the output port of the HPF. The HPF has maximum matching near 5 GHz. Both the filters are designed such that they have high insertion loss on one another’s region to ensure proper operations of the antennas connected at their output ports. The design details of the communicating antenna and the sensing antenna are discussed below.

### Design of fixed band communicating antenna

The proposed fixed band communicating antenna (FBCA) is designed to operate below the cut-off frequency of the LPF as it is connected to the output port of the LPF of FSMF. The FBCA is designed based on a modified monopole^30^ with a T-shape. The design details of the proposed FBCA are shown in Fig. 5, where, $A_{L1}=8.1$, $A_{L2}=18$, $A_{L3}=15$, $A_{L4}=8$, $A_{L5}=8.8$, $A_{L6}=4$, $A_{L7}=6.5$, $A_{L8}=4$, $A_{W1}=1.77$, $A_{W2}=5$, $A_{G1}=5.8$, $a=20.4$, $b=12.1$, $c=42.2$, $d=26$, $e=86.8$, and $f=20$ (all in mm). The size of the ground plane, substrate, and feedline’s thickness of the FBCA has been kept the same as the complete proposed system shown in Fig. 3a to ensure minimum reflection upon integration with the FSMF. The input reflection coefficient of the proposed FBCA is shown in Fig. 6a. The FBCA has a −10 dB impedance bandwidth of 2–3 GHz. The radiation pattern of the FBCA at 2.45 GHz for phi=0$^{\circ }$ and phi=90$^{\circ }$ planes is shown in Fig. 6b and c.

**Figure 7:**
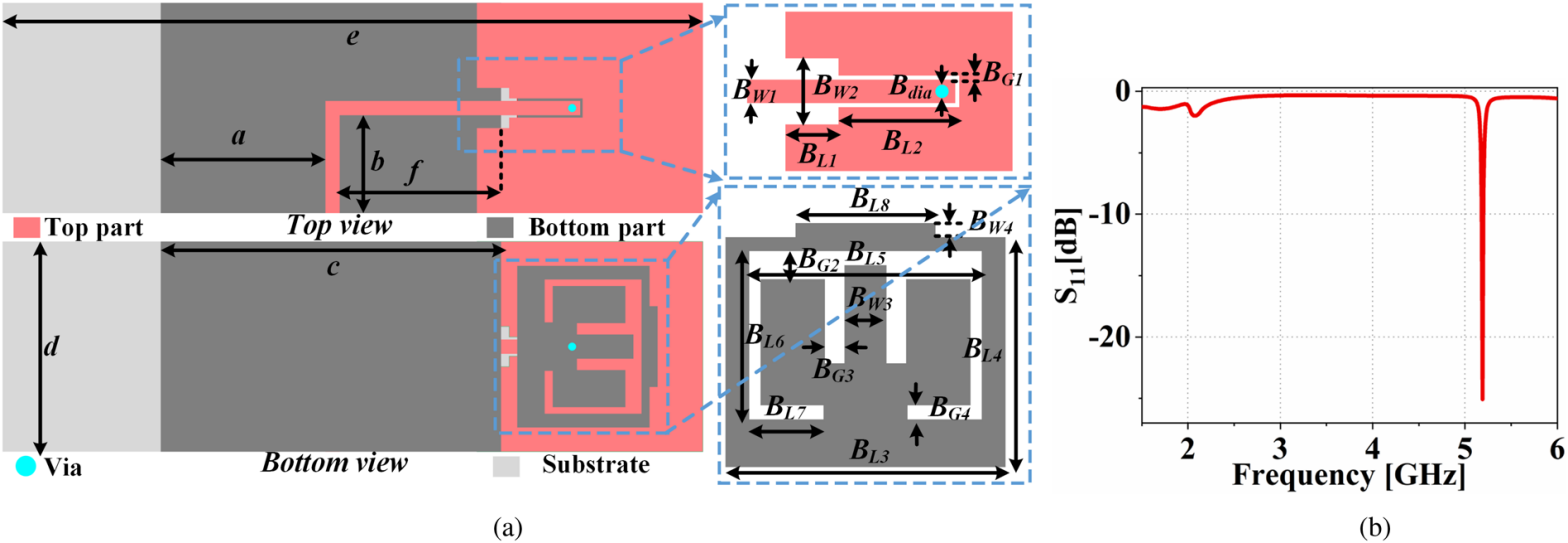
(**a**) Proposed antenna for sensing application. (**b**) Input reflection coefficient of the proposed antenna sensor.

### Design of antenna sensor

The proposed antenna sensor is designed to operate above the cut-off frequency of the HPF as it is connected to the output port of the HPF of FSMF. The design details of the antenna sensor are shown in Fig. 7a, where, $B_{W1}=1.77$, $B_{W2}=5$, $B_{W3}=3$, $B_{W4}=1$, $B_{L1}=4$, $B_{L2}=9.1$, $B_{L3}=20$, $B_{L4}=16.4$, $B_{L5}=16.6$, $B_{L6}=12$, $B_{L7}=5.3$, $B_{L8}=10$, $B_{G1}=0.3$, $B_{G2}=2$, $B_{G3}=1.4$, $B_{G4}=1$, $B_{dia}=1$, $a=20.4$, $b=12.1$, $c=42.2$, $d=26$, $e=86.8$, and $f=20$ (all in mm).The proposed antenna sensor has two patches, one parasitic patch (patch-1) on the top side and another patch (patch-2) on the bottom side. The bottom patch is connected to the main feedline through via. The radiating patch of the FBCA lies on the top part of the proposed system, as shown in Fig. 3a. Therefore, to have a different sensing plane, one patch of the antenna sensor is kept at the other side of the PCB. The antenna sensor’s substrate and ground plane have the same dimensions as the complete proposed system shown in Fig. 3a. In addition, the thickness of the main feed line is also kept the same as the output port of the HPF of FSMF to ensure minimum reflection upon integration. The input reflection coefficient of the antenna sensor is shown in Fig. 7b. The antenna sensor resonates at 5.19 GHz. The resonance frequency of the antenna sensor will shift upon perturbation of the electric field. The lower limit of the frequency shift is limited due to the cut-off frequency of the HPF. Hence, the antenna sensor is designed such that without any perturbation of the electric field it resonates at frequency away from cut-off frequency of the HPF. The unperturbed resonance frequency of the antenna and cut off frequency of the HPF will decide the range of frequency shift.

**Figure 8:**
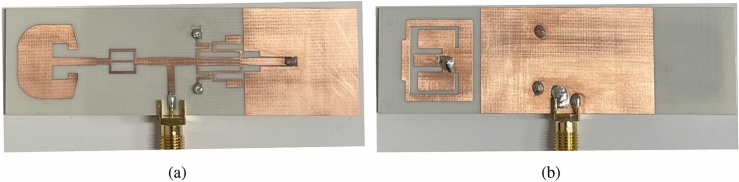
Fabricated prototype of the proposed dual-functional antenna system. (**a**) Top view, (**b**) bottom view.

**Figure 9:**
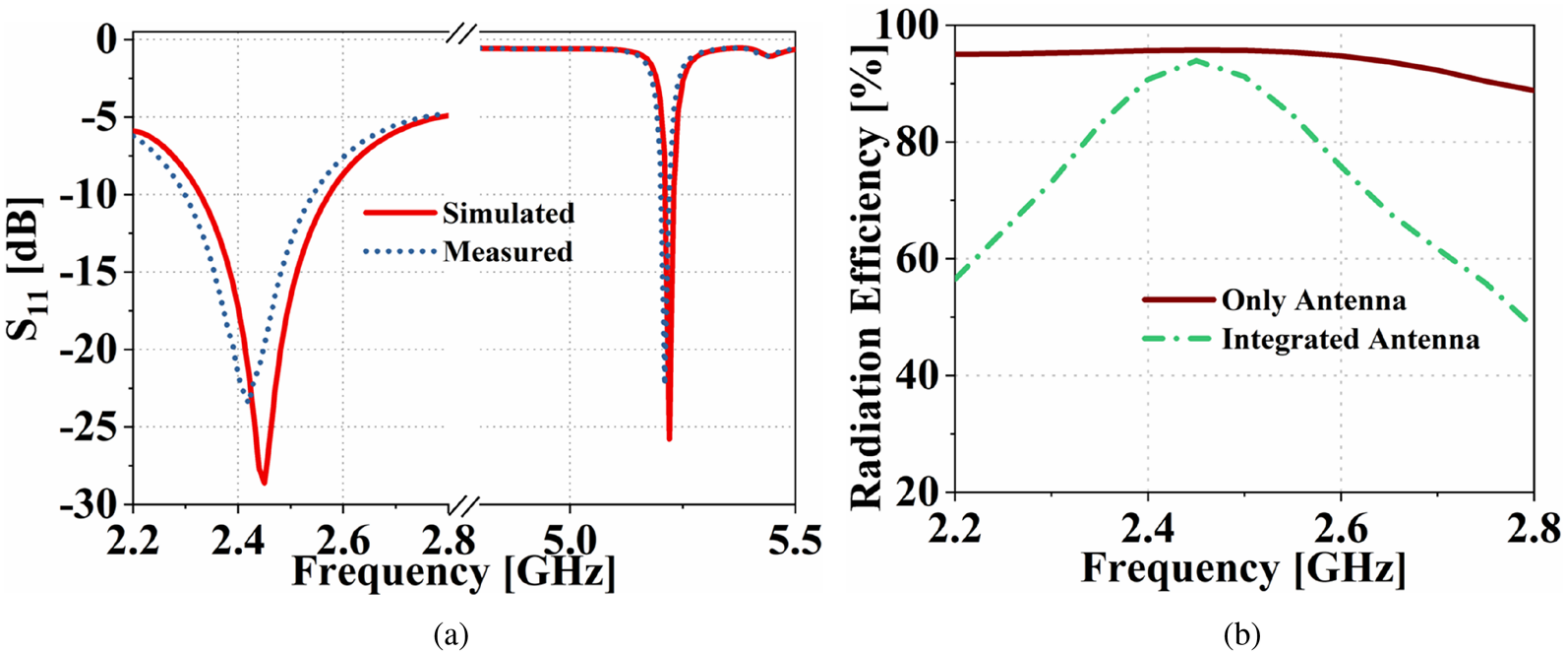
(**a**) The simulated and measured input reflection coefficient of the proposed dual-functional system. (**b**) Radiation efficiency of the the communication antenna before and after integration with the complete design.

## Results and discussion

**Figure 10:**
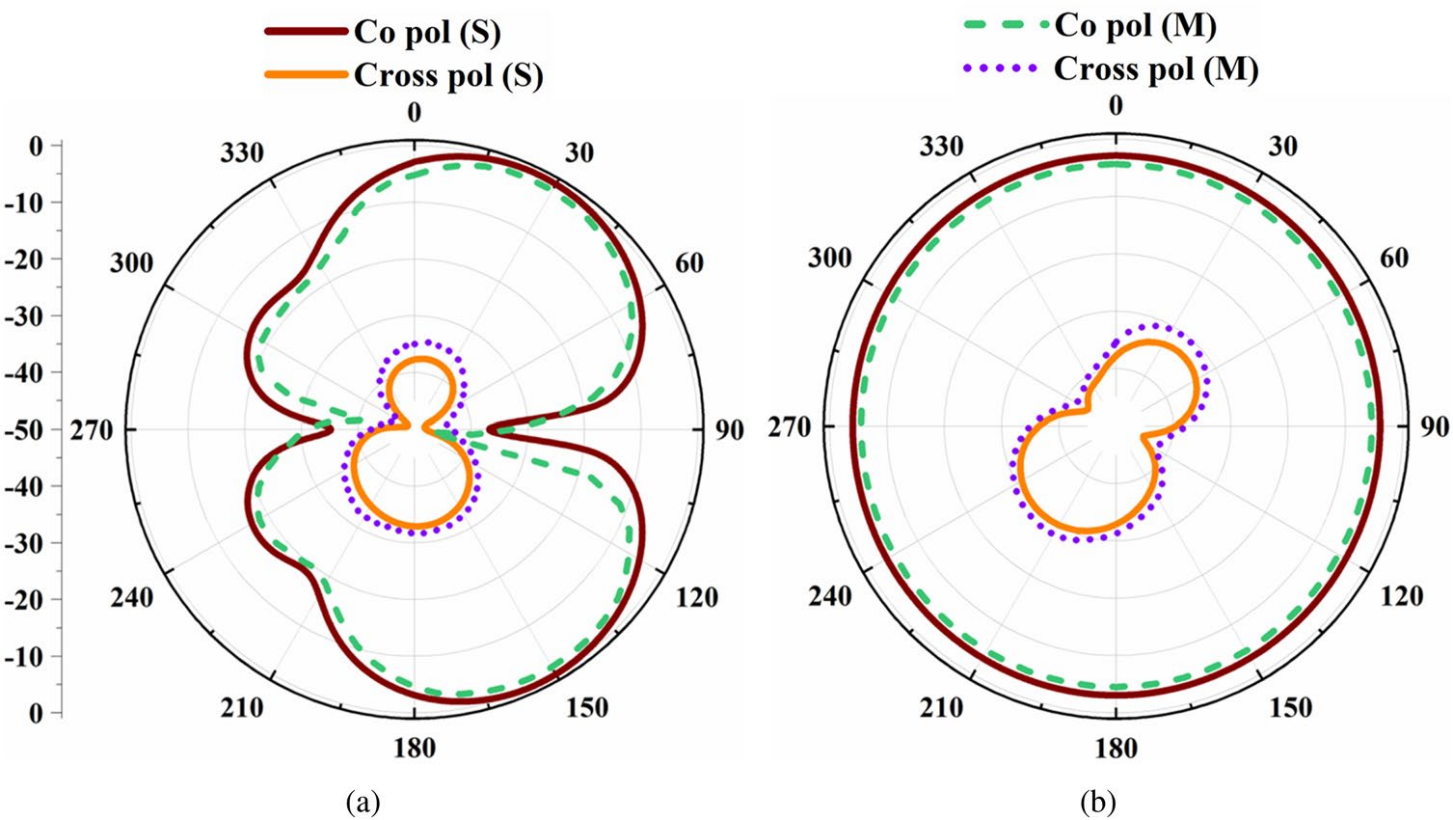
The simulated and measured radiation pattern of the FBCA at 2.45 GHz in (**a**) $\hbox {phi}=0^{\circ }$, (**b**) $\hbox {phi}=90^{\circ }$ planes.

The FBCA and the antenna sensor are integrated together using the FSMF, as shown in Fig. 3a. After integration with the FSMF, the resonance frequency of the antenna sensor shifted from 5.19 GHz to 5.22 GHz. Similarly, integration of the LPF with the FSMF reduces the matching of the filter. Hence, after integrating the FBCA with FSMF, the −10 dB impedance of the communicating antenna gets reduced from 2–3 GHz to 2.33–2.56 GHz. The proposed dual-functional antenna system’s fabricated prototype is shown in Fig. 8. The simulated and measured input reflection coefficient of the proposed dual-functional system is shown in Fig. 9a. The simulated radiation efficiency of the FBCA before and after integrating with the complete system is shown in Fig. 9b. After integration, the radiation efficiency of the FBCA at 2.45 GHz gets reduced due to the insertion loss of the LPF. The measured radiation efficiency of the antenna is 88% at 2.45 GHz. The measured gain of the proposed dual-functional system at 2.45 GHz is 3.5 dB. The simulated and measured radiation pattern of the FBCA of the dual functional system at 2.45 GHz at $\hbox {phi}=0^{\circ }$, and $\hbox {phi}=90^{\circ }$, planes are shown in Fig. 10. The simulated and measured results are in good agreement.

**Figure 11:**
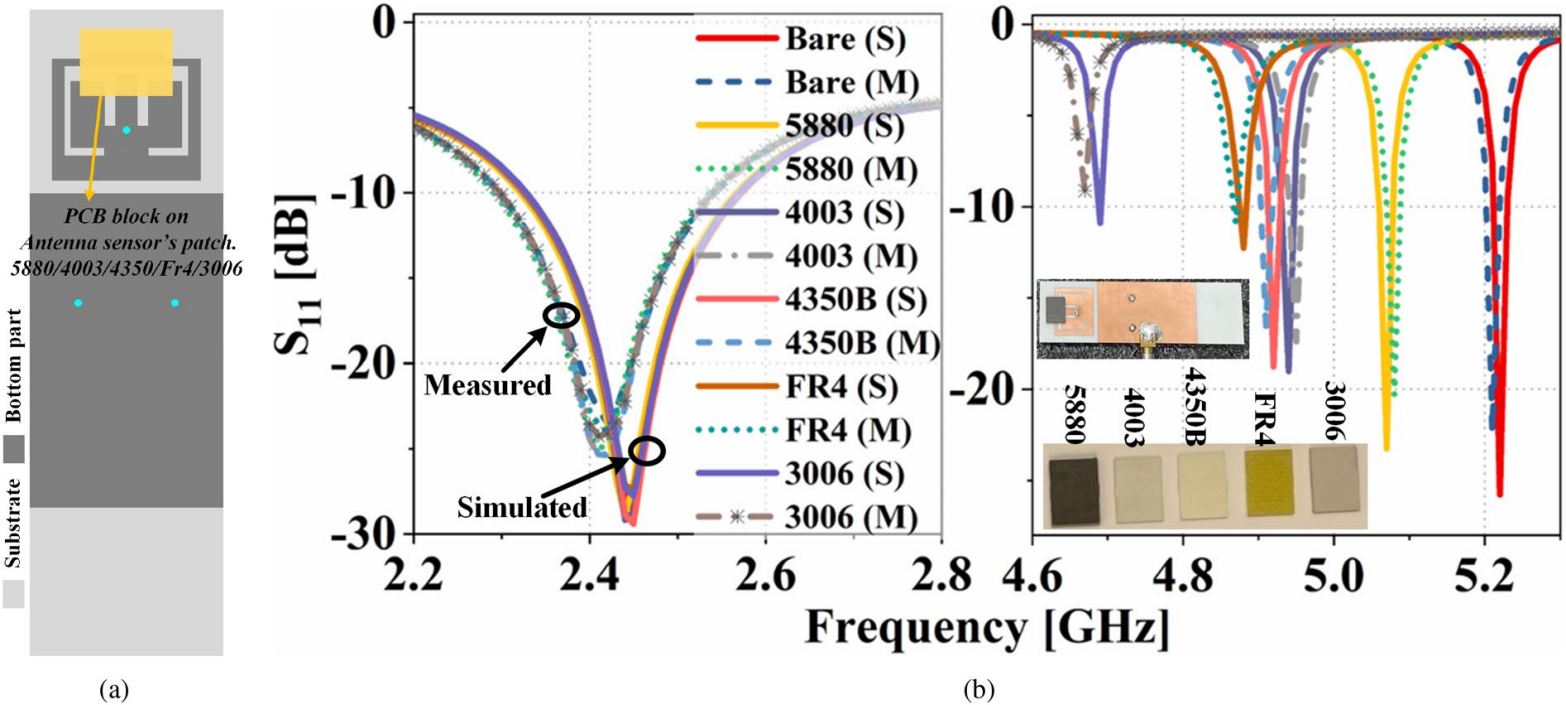
(**a**) Substrate block on top of the antenna sensor. (**b**) Simulated and measured Input reflection coefficients of the proposed system in presence of different substrate blocks on the antenna sensor.

**Figure 12:**
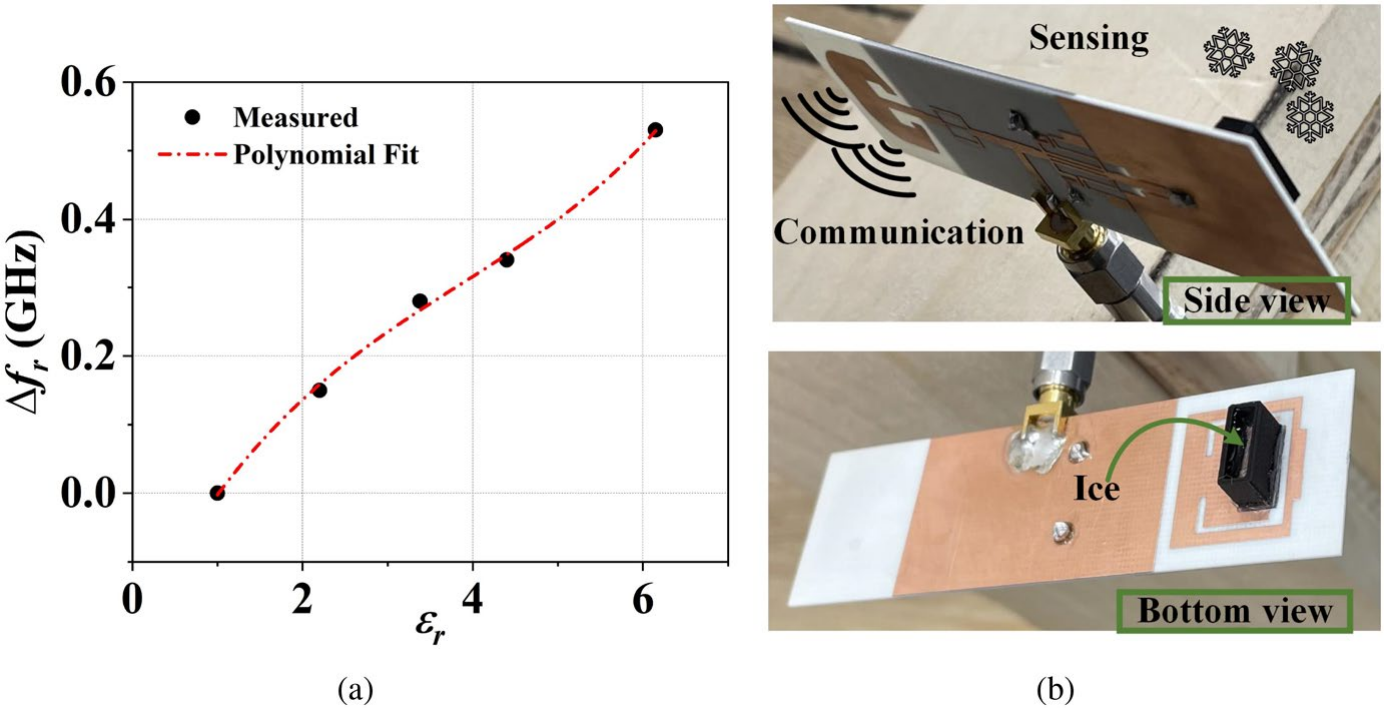
(**a**) The measured and polynomial fit of the resonance frequency shifts of the antenna sensor with respect to the permittivity of the MUTs. (**b**) Sensing and communication plane of the dual-functional antenna system.

The performance of the proposed dual-functional antenna system is first validated by sensing different substrate materials (PCB blocks: Rogers RT/duroid 5880, RO 4003C, RO 4350B, Fr4, and RO 3006) of dimension $10.5\times 8.5\,\text {mm}^2$. The substrates have dielectric constant from a range of 2.2 to 6.15. The measurements are done by directly keeping each substrate block on top of the antenna sensor’s patch-2, as shown in Fig. 11a.

The top and bottom patches of the antenna sensor together act as a cavity. Hence, the resonance frequency shift of the antenna sensor due to perturbation of electric field in the presence of a substrate block can be defined as^31^

1 $$\begin{aligned} \left( \Delta {f_{r}}/f_r\right) = -\left( \frac{{\varepsilon} _{substrate}-1}{2}\right) \left( \frac{\int _{V_s}{E}\cdot {E_{perturbed}}dV}{\int _{V_c}|E_{perturbed}|^2dV}\right) \end{aligned}$$

**Figure 13:**
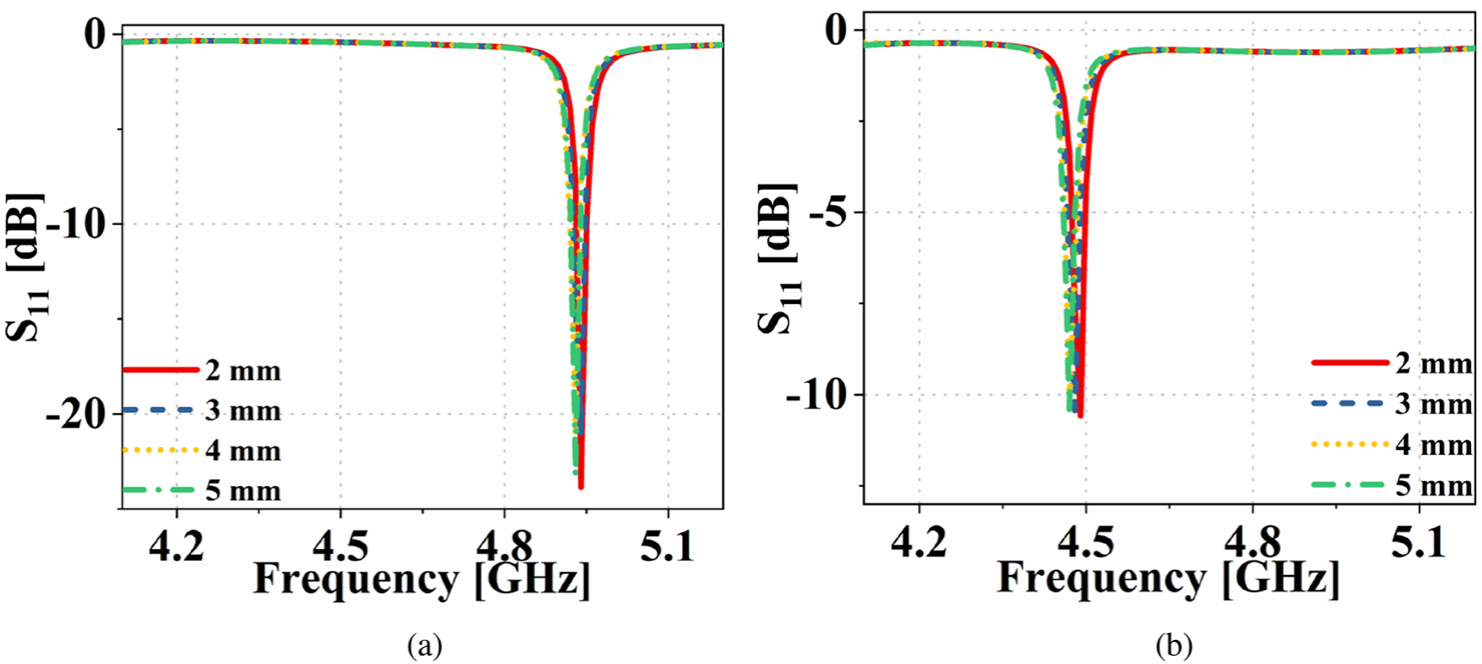
Input reflection coefficient of the dual-functional antenna system in presence of different height of (**a**) ice and (**b**) water inside the container.

**Figure 14:**
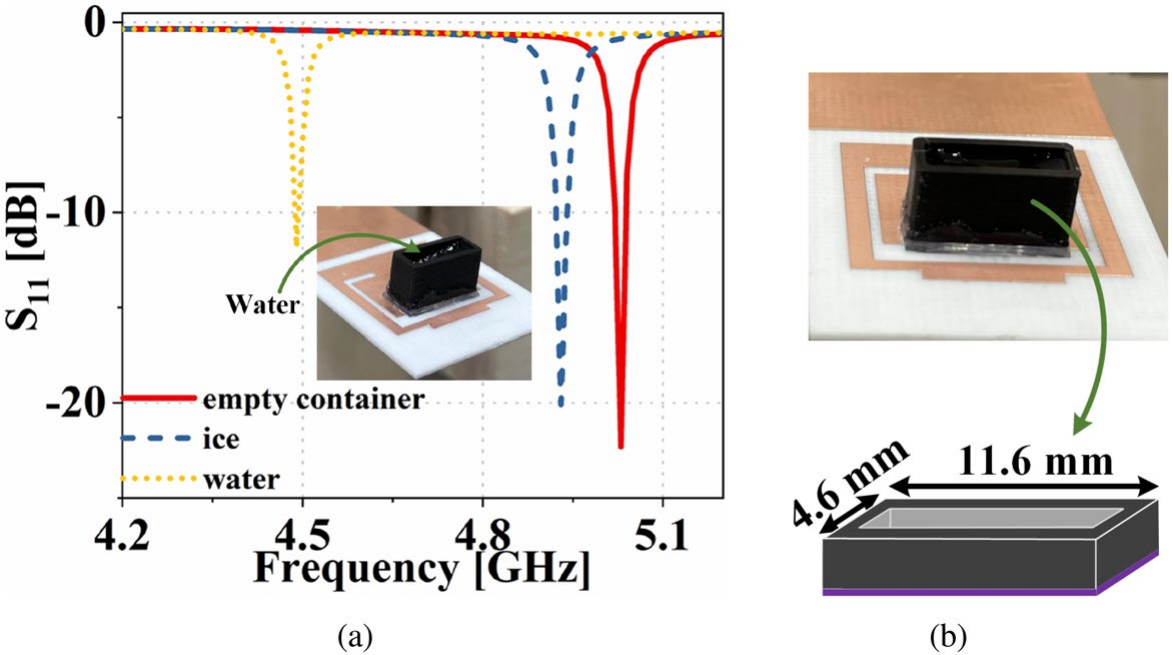
(**a**) Measured input reflection coefficients of the dual functional antenna system in presence of empty container, ice and water, (**b**) container.

where, $\Delta {f_{r}}$ is the resonance frequency ($f_r$) shift in the presence of a substrate block. *E* is the electric field (unperturbed) of the bare antenna sensor, and $E_{perturbed}$ is the electric field in the presence of a substrate block. Due to different ${\varepsilon} _r$, each substrate block perturbs the electric field differently, shifting the antenna sensor’s resonance frequency. The simulated and measured input reflection coefficients shown in Fig. 11b confirm this. The simulated and measured results are in good agreement. The input reflection coefficients of Fig. 11b show that the proposed sensor resonates at 4.94 GHz for RO 4003c (${{\varepsilon}}_r=3.38$) and 4.92 GHz for RO 4350B (${\varepsilon} _r=3.48$). Hence, the proposed dual-functional antenna system can be useful for distinguishing two different materials with a very small deviation in electrical permittivity value. Furthermore, Fig. 11b also shows that the operational frequency band of the communicating antenna is almost unaffected despite the presence of different materials on the antenna sensor. This shows that our proposed single-port dual-functional system can be useful for communicating and sensing applications without compromising each other’s performance. The measured resonance frequency shift of the antenna sensor and its polynomial fit in the presence of different MUTs are presented Fig. 12a. As per the polynomial fit, the unknown permittivity of a MUT with respect to its resonance frequency shift ($\Delta f_{r}$) can be derived as

2 $$\begin{aligned} {\varepsilon} _r(\Delta {f_{r}}) = -27.34\cdot (\Delta {f_{r}})^3+24.57 \cdot (\Delta {f_{r}})^2+4.36\cdot (\Delta {f_{r}})+1 \end{aligned}$$

The antenna sensor is also tested for ice/water detection applications. the communication plane and sensing plane of the dual-functional antenna system is shown in Fig. 12b. Rather than directly applying ice/water on top of the sensor’s patch, a 3D printed container using polyactic acid (PLA) (${\varepsilon} _r=2.6$ and $\tan \delta =0.025$) is used. The container is glued with a polypropylene sheet (${\varepsilon} _r=2.25$ and $\tan \delta =0.006$) of 0.5 mm thickness. The polypropylene sheet acts as a bottom wall of the container. Therefore, ice/water in the container is separated from the antenna sensor by $50\,\mu {m}$. The detailed dimensions of the container are given in Fig. 14b.

Ice has an ${\varepsilon} _r=3.48$ and $\tan \delta =0.0009$^32^. The simulated input reflection coefficients of the antenna sensor for different heights of ice are shown in Fig. 13a. Similarly, the simulated input reflection coefficient for different heights of water inside the container is shown in Fig. 13b. The figure shows that the resonance frequency of the antenna sensor remains almost the same for different heights of ice/water. Hence the proposed sensor can be an ideal candidate for distinguishing different liquid/solids despite the change in their volume/height. Being insensitive to the height of the MUT, the sensor also eliminates human errors, which can be made by filling more/less liquids during e.g., lab experiments for detecting different liquids. The ice sample for measurement is prepared by keeping the container containing water inside a refrigerator, and the measurements are done immediately after taking out the container from the refrigerator. The measured input reflection coefficient of the antenna sensor, in the presence of an empty container, with ice and water is shown in Fig. 14a. Here it is evidenced the ability to detect the different cases.

## Conclusions

In this paper, a single port dual-antenna system for simultaneous operation of communication and sensing is presented. The system includes an antenna for a fixed-band communication and a different antenna for sensing purposes. Both the antennas are integrated such that they work as a single port device. The main objective of the proposed system is to detect different material under tests (MUTs) without affecting the operational bandwidth of the communicating antenna. To achieve this, a frequency selective multipath filter (FSMF) is designed. The FSMF has an input port and two output ports. The antennas are integrated on each output port of the FSMF. The performance of the proposed dual-functional system is validated using different PCB substrate blocks and in the presence of ice/water. The simulated and measured results are in good agreement. The measured results show that the presence of MUTs on the antenna sensor only shifts the resonance frequency of the antenna sensor. The operational bandwidth of the communicating antenna remains unaffected.

## Data Availability

All data generated or analyzed during this study are included in this manuscript.
